# Effective Multi-agent Therapy With Anakinra, Corticosteroids and Mycophenolate in Immunotherapy-Associated Haemophagocytic Lymphohistiocytosis (HLH)

**DOI:** 10.7759/cureus.111913

**Published:** 2026-07-01

**Authors:** Anirban Deb Tanmoy, Fan Jiong Eaw, Charlotte Mykura, Dhanush Sheeba Pradeep, Samantha Gold

**Affiliations:** 1 Internal Medicine, Northampton General Hospitals NHS Trust, Northampton, GBR; 2 Oncology, University Hospitals Dorset NHS Foundation Trust, Bournemouth, GBR

**Keywords:** anakinra treatment, cancer-immunotherapy, haemophagocytic lymphohistiocytosis (hlh), immune-checkpoint inhibitor adverse effects, mycophenolate mofetil (mmf)

## Abstract

Haemophagocytic lymphohistiocytosis (HLH) is a rare, life-threatening complication of immune checkpoint inhibitor (ICI) therapy. Clinical overlap with sepsis contributes to delayed diagnosis and high mortality, showing the need for prompt recognition and targeted immunosuppression. This case presents a successful treatment pathway for HLH with high-dose methylprednisolone, mycophenolate mofetil (MMF) and anakinra, as well as emphasising the importance of early detection and management of HLH.

A man in his sixties with stage IV cutaneous melanoma presented six weeks after the first cycle of ipilimumab and nivolumab immunotherapy with persistent high-grade fever, rigours, myalgia, malaise, and weight loss. Initial investigations revealed no infectious source, but computed tomography (CT) scans showed splenomegaly, and laboratory findings, including high ferritin, hypertriglyceridemia, high aspartate aminotransferase (AST), and low cell counts in two lines (lymphopenia and thrombocytopenia), met the modified HLH-2009 diagnostic criteria within 72 hours. Absence of melanoma progression, infection, or genetic predisposition suggested immunotherapy as the trigger. Treatment commenced on day 1 with anakinra and high-dose intravenous methylprednisolone, with mycophenolate mofetil added on day 3 due to worsening transaminitis, indicative of concurrent immunotherapy-induced hepatitis. The patient became afebrile within 48 hours, with ferritin normalising by day 10 and haematological and biochemical markers returning to normal. Anakinra was discontinued after 14 days. Complications included steroid-induced hyperglycemia, herpes simplex infection, and insomnia, all managed medically. At one-month follow-up, the patient remained asymptomatic with no HLH recurrence during immunosuppression tapering. This case highlights that steroids remain the primary acute treatment for HLH and immune-mediated disorders, with anakinra effectively inhibiting Interleukin-1 driven cytokine overproduction and mycophenolate mofetil addressing immune-related hepatitis. The combination of anakinra, mycophenolate mofetil, and high-dose methylprednisolone achieved rapid clinical and biochemical remission within 10 days, consistent with adult HLH consensus guidelines advocating early, multi-agent immunosuppression to prevent relapse in hyperinflammatory syndromes. Close monitoring of HLH biomarkers and a low threshold for suspicion in febrile patients on immunotherapy is recommended to facilitate timely intervention.

## Introduction

Haemophagocytic lymphohistiocytosis (HLH) is a rare hyperinflammatory disorder characterised by uncontrolled activation of histiocytes and lymphocytes, resulting in the immune-mediated haemophagocytosis of haematological cells [1]. HLH can be broadly classified into two categories: primary HLH through inherited immunodeficiency syndromes or inborn errors of immunity, usually occurring in children; and secondary HLH acquired through infection, malignancy or as a result of autoimmune conditions, usually occurring in adults [2].

This case presents a patient treated with ipilimumab and nivolumab who developed secondary HLH with transaminitis. Combined ipilimumab (anti-CTLA-4) and nivolumab (anti-PD-1) is a dual immune-checkpoint inhibitor (ICI) regimen that is approved and widely used as first-line therapy for unresectable or metastatic melanoma, and that significantly improves overall survival compared with single-agent therapy [3].

Secondary HLH as an adverse effect of ICIs is rare and therefore not well characterised; a recent literature review has shown that between 2017 and 2023, there were only 51 cases of ICI-induced HLH [4]. This may be due to the high mortality rate of HLH, with one study reporting 42% [5]. However, clinical overlap with sepsis and resultant end-organ dysfunction likely also leads to the underdiagnosis of HLH.

Of reported HLH cases, some medical interventions have proved effective, for example, with steroids only or with steroids and tocilizumab [6,7]. Recommended HLH treatments have classically been developed from paediatric HLH protocols, such as the HLH-94 protocol, which recommended dexamethasone, etoposide and cyclosporin A [8].

In this case, successful treatment was achieved with a combination of high-dose methylprednisolone, mycophenolate mofetil (MMF) and anakinra with minimal adverse effects. This case presents a novel treatment pathway for HLH, as well as emphasising the importance of early detection and management of HLH.

## Case presentation

This case presents a Caucasian male patient in his early 60s with a history of cutaneous melanoma on the back, diagnosed 5 years prior. His melanoma was diagnosed at stage III, treated with wide local excision first and then four years down the line, he had recurrence in the right flank with right axillary and subcutaneous deposits, as well as extensive abdominopelvic mesenteric metastatic nodal involvement, which was stage four. Then, ipilimumab and nivolumab were considered for him. The patient had no other significant past medical history and no documented personal or family history of autoimmune or hematologic disorders. He was a non-smoker and reported occasional social alcohol consumption.

He presented with a fever persisting for three days, accompanied by rigours, myalgia, and general malaise. The highest recorded temperature was 39.9 °C. He also reported occasional night sweats and a weight loss of around 12 kilograms in the last 6 months. There was no abdominal or chest pain, no shortness of breath, nor cough. He reported normal urinary function. Although he had experienced a loss of appetite, he continued to consume small meals. On the day of presentation, the patient was one month post the first cycle of his ipilimumab and nivolumab therapy, having not had any prior complications of the treatment.

On examination, he was alert, comfortable and haemodynamically stable. There was no evident jaundice or anorexia. Auscultation of the chest revealed clear lung fields without additional sounds, and he had a soft and non-tender abdomen with no obvious organomegaly. His calves were soft and non-tender. Apart from high temperature, he was normotensive, with normal oxygen saturation on room air, and slightly tachycardic. The initial sepsis screening, including blood and urine cultures and a chest X-ray, was unremarkable. The patient was thus treated empirically as a case of pyrexia of unknown origin with broad-spectrum antibiotics.

A computed tomography scan of the chest, abdomen, and pelvis (CTCAP) was conducted to exclude disease progression or new metastasis, which the radiologists had initially reported as no acute findings and stable cancer. Initial blood tests indicated lymphopenia and thrombocytopenia; ferritin, aspartate aminotransferase (AST) and triglyceride levels were elevated (Table 1).

**Table 1:** Initial blood tests done for the patient leading to the diagnosis of HLH HLH: haemophagocytic lymphohistiocytosis; eGFR: estimated glomerular filtration rate; ALT: alanine transaminase; AST: aspartate transferase; /L: per litre; g/L: gram per litre; µg/L: micrograms per litre; mg/L: milligrams per litre; mL/min/1.73 m²: millilitre per minute per 1.73 square meter; mmol/L: millimoles per litre; U/L: units per litre; µmol/L: micromoles per litre Values in bold are the abnormal values leading to the diagnosis of HLH in the patient.

Blood Investigations	Values	Reference Ranges
Total white cell count (10*9/L)	4.3	4.0 - 10.0
Lymphocyte count (10*9/L)	0.4	1.0 - 3.0
Neutrophil count (10*9/L)	3.6	2.0 - 7.0
Haemoglobin estimation (g/L)	126	130 - 170
Platelet count (10*9/L)	118	150 - 410
Serum ferritin (µg/L)	656	30 - 400
Fibrinogen level (g/L)	4.08	1.88 - 4.15
International normalised ratio	1	0.9 - 1.2
Serum C-reactive protein level (mg/L)	36	0 - 9
Serum creatinine (µmol/L)	88	59 - 104
eGFR (mL/min/1.73m*2)	81	>90
Serum potassium (mmol/L)	4.1	3.5 - 5.0
Serum sodium (mmol/L)	132	132 - 146
Serum urea level (mmol/L)	6	2.5 - 6.7
Serum ALT level (U/L)	203	0 - 35
Serum total bilirubin level (µmol/L)	11	0 - 17
AST serum level (U/L)	124	0 - 35
Serum triglycerides (mmol/L)	2.4	0.5 - 2.3

**Figure 1:**
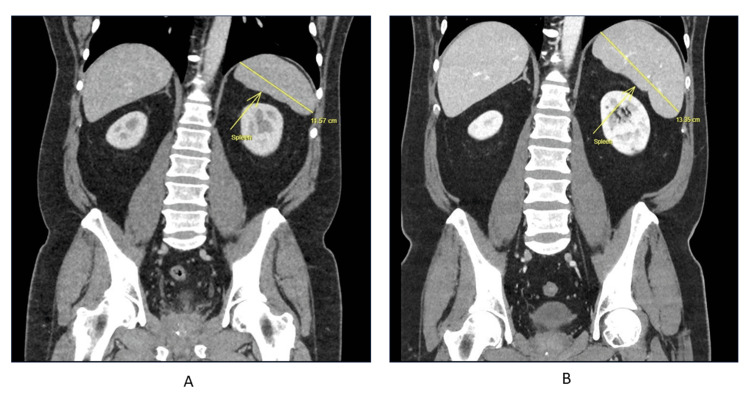
Image A: CT scan showing the spleen (11.57 cm) of the patient one year ago; Image B: CT scan showing the size of the spleen (13.35 cm) at the time of diagnosis of HLH HLH: haemophagocytic lymphohistiocytosis; CT: computed tomography, cm: centimetres

Due to suspicion of HLH, the CTCAP was re-reviewed, which showed a subtly enlarged spleen (Figure 1).

These findings met the 2009 modified diagnostic criteria for HLH, characterised by lymphopenia, thrombocytopenia, elevated ferritin, splenomegaly and hypertriglyceridemia [9]. Microbiological screening for viral hepatitis and latent tuberculosis was negative, as was the non-invasive liver screen. At the time of diagnosis, the Hscore (score for assessing the probability of haemophagocytic syndrome) was 159, and treatment was started due to high suspicion of HLH (Table 2) [10].

**Table 2:** Hscore assessment for HLH HLH: hemophagocytic lymphohistiocytosis; °C: degrees Celsius; mmol/L: millimoles per litre; g/L: grams per litre; AST: aspartate aminotransferase; U/L: units per litre; ng/mL: nanograms per millilitre This table details the individual parameter scoring for the assessment of reactive hemophagocytic syndrome (HScore). The total score is calculated by summing the points from each category to estimate the overall probability of HLH.

Parameter	Score Criteria	Patient Value
Known underlying immunosuppression	No (0), Yes (18)	No (0)
Temperature (°C)	<38.4 (0), 38.4–39.4 (33), >39.4 (49)	39.9 (49)
Number of Cytopenias	1 lineage (0), 2 lineages (24), 3 lineages (34)	2 lineages (24)
Hepatomegaly/Splenomegaly	No (0), Hepatomegaly or Splenomegaly (23), Hepatomegaly and Splenomegaly (38)	Yes (23)
Triglycerides (mmol/L)	<1.5 (0), 1.5–4.0 (44), >4.0 (64)	3.4 (44)
Fibrinogen (g/L)	>2.5 (0), ≤2.5 (30)	4.0 (0)
AST (U/L)	<30 (0), ≥30(19)	135 (19)
Ferritin (ng/mL)	<2000 (0), 2000–6000 (35), >6000 (50)	600 (0)
Hemophagocytosis features on bone marrow aspirate	No (0), Yes (35)	Not done (0)
Total Hscore		159

Initial treatment comprised anakinra, a modified recombinant version of the human interleukin 1 (IL-1) receptor antagonist protein, which was started at 4 mg/kg. This treatment was chosen specifically due to the report IL-1-driven nature of HLH [11]. Escalation doses were planned if there was no clinical or haematological response. Concurrently, 1 g of intravenous methylprednisolone was given.

The patient responded well and remained apyrexial after starting anakinra. By the third day, liver function tests (LFTs) exhibited an upward trend, raising concerns regarding potential immunotherapy-related hepatitis. Anakinra hepatotoxicity was also considered; however, reported cases of this are extremely rare (<1%) in large registration trials [12]. Thus, it was considered, on balance, to continue the anakinra. LFTs were monitored closely.

**Figure 2:**
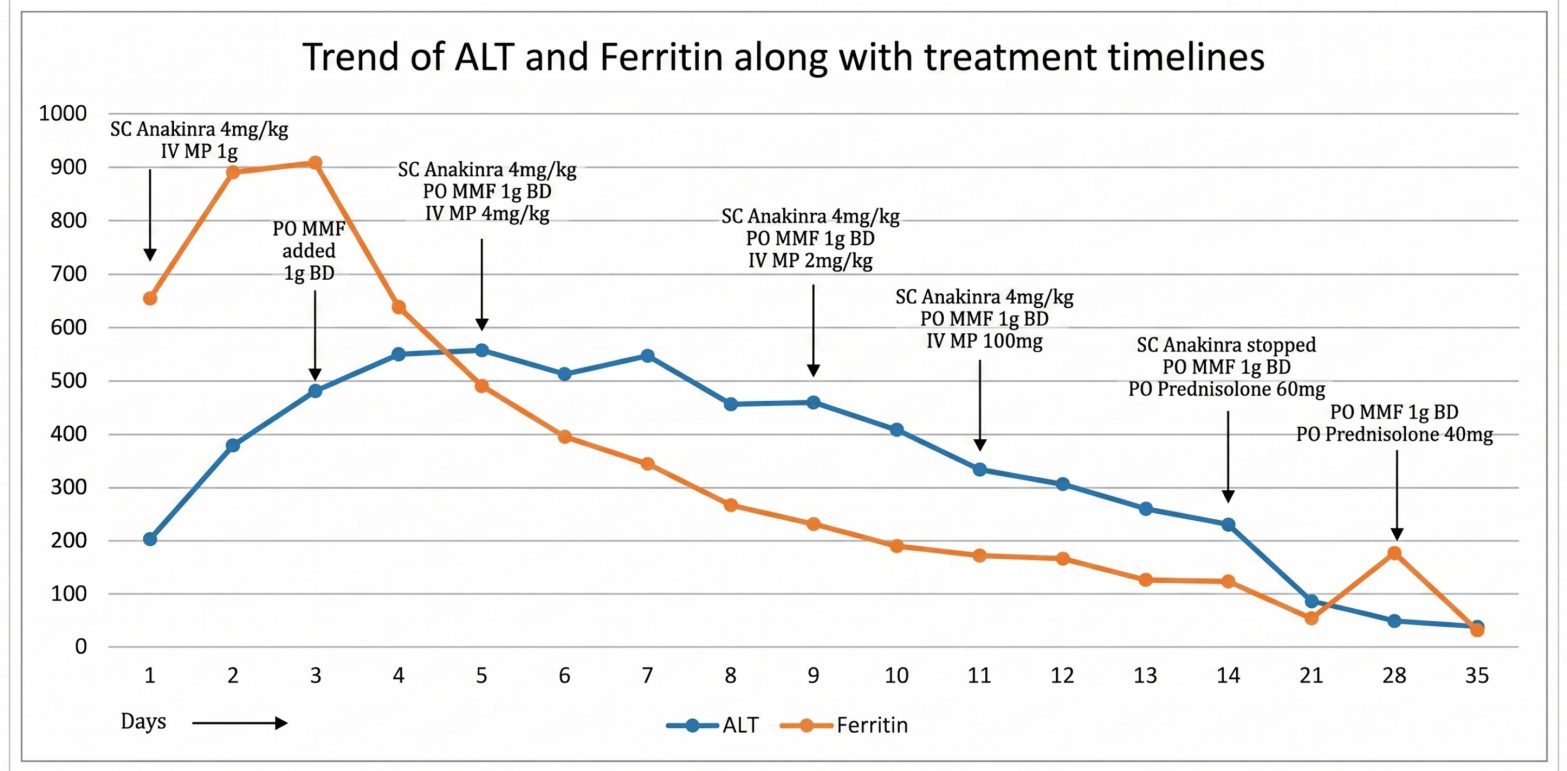
Trends in ALT (U/L) and ferritin (µg/L) levels after treatment initiation and subsequent dose adjustments ALT: alanine aminotransferase (U/L); BD: bis in die (twice a day); IV: intravenous; MMF: Mycophenolate Mofetil; MP: methylprednisolone; PO: per oral; SC: subcutaneous; g: gram; mg: milligram; U/L: units per litre; µg/L: micrograms per liter; mg/kg: milligrams per kilogram Both ferritin and ALT are plotted against a single reference axis, as their clinical values naturally shared a matching numerical scale (0–1000), intended here to highlight simultaneous chronological trends rather than direct value comparisons.

Consequently, 1 g of mycophenolate mofetil (MMF) was administered twice daily orally in addition to the existing treatment regimen to treat possible immuno-oncology (IO) hepatitis. Subsequent blood tests indicated a progressive improvement, with ferritin levels returning to normal and LFTs showing a 50% improvement by the 10th day (Figure 2).

The patient was clinically stable. Notable side effects of the treatment included steroid-induced insomnia, hyperglycaemia, upper lip cold sores indicative of herpes simplex infection and mouth ulcers, which were managed with appropriate medical treatment.

After 5 days of 1 g once daily methylprednisolone, this was reduced to 4 mg/kg. On day 9 of treatment, methylprednisolone was reduced further to 2 mg/kg and further reduced to 100 mg methylprednisolone on day 11. On day 14, the anakinra was discontinued, and corticosteroids were switched to an oral weaning dose of prednisolone. The patient was discharged home on day 15 on 1 g of MMF twice daily and 60 mg of oral prednisolone to be weaned in the community. He was referred to the immunotherapy toxicity clinic, which followed him up weekly. At the one-week follow-up, the patient had remained apyrexial and was systemically well, but noted experiencing ongoing muscle fatigue, insomnia and mouth ulcers. This was treated with conservative measures. At the one-month follow-up, the patient reported feeling well with no new symptoms. Following the successful tapering of steroids and the maintenance of clinical stability for a duration of two weeks, the dosage of MMF was reduced to 500 mg administered twice daily for one week. Subsequently, the medication was discontinued after an additional week. Blood tests on follow-up showed resolved LFTs, cell counts and ferritin levels.

## Discussion

Immunotherapy-associated HLH remains rare but is increasingly recognised in patients receiving anti-PD1 and CTLA-4 combination therapy such as ipilimumab and nivolumab. Another reported case has also demonstrated HLH presenting in a patient treated with cabozantinib along with ipilimumab and nivolumab, showing that HLH may develop in patients on other immunotherapy regimens and even targeted therapies [13].

HLH presents similarly to sepsis, with patients commonly presenting with pyrexia, malaise and raised inflammatory markers. This clinical overlap can lead to a delayed diagnosis of HLH, increasing mortality rates by up to 30-40% and consequently leading to the underdiagnosis of HLH, despite fulfilling the HLH criteria [14]. There are also diagnostic challenges, as early HLH findings such as hepatomegaly or splenomegaly may be subtle on imaging, as seen in this case.

The diagnosis of HLH was facilitated by early clinical suspicion, allowing for rapid re-reviewing of existing imaging and biomarker assessment. The absence of infective foci, disease progression, or genetic predisposition strongly implicated ipilimumab/nivolumab as the trigger. The timing was also consistent with cases reported, where HLH onset was 4-12 weeks post-systemic anti-cancer therapy [6,7,15]. Our patient developed symptoms 6 weeks post-immunotherapy, but diagnostic confirmation occurred in less than 72 hours post-presentation due to early detection of splenomegaly and rapid biomarker review.

Steroids remain the primary acute treatment for any HLH or immune-mediated disorders due to their ability to rapidly suppress the immune system. However, monotherapy with steroids may be insufficient in secondary HLH. In a study of 22 patients with secondary HLH, even though all patients received corticosteroids, some patients required additional immunosuppressants, for example, etoposide (23%), intravenous immunoglobulin (5%), cytokine inhibitors, such as tocilizumab or anakinra (23%), or immunosuppressants such as MMF, tacrolimus or cyclosporine (14%). HLH resolved in 19 of the 22 patients, where different immunosuppressant combinations were used, demonstrating the importance of early intervention and efficacy of combination therapies [16].

Anakinra has been shown to be an effective and well-tolerated treatment in HLH. This is because HLH is characterised by rapid, uncontrolled cytokine release from hyperactivated macrophages, with IL-1 being overexpressed, as shown by gene profiling of peripheral blood mononuclear cells in active HLH [17]. According to a multicentric retrospective study, anakinra demonstrated high effectiveness in treating secondary HLH, with 90.5% of patients showing clinical improvement, including fever resolution within a median of 1 day, alongside reductions in mean CRP and ferritin levels by Day 7 [11,18]. This finding aligned with the biochemical response to treatment observed in our case. Our patient received 4mg/kg/day and had minimal adverse effects, which were consistent with the recommended dose of 1 to 8 mg/kg/day.

However, our patient’s LFTs worsened within three days of commencing anakinra, possibly anakinra-induced. However, in a systematic review of 87 patients, adverse reactions to anakinra were infrequent with minimal reports of transaminitis, indicating a generally favourable safety profile for intravenous or subcutaneous anakinra in the treatment of HLH and that this LFT rise was likely concurrent immunotherapy-associated hepatitis [19].

MMF was added due to worsening transaminitis in the context of suspected simultaneous immunotherapy-associated hepatitis. Both the American Society of Clinical Oncology (ASCO) and European Society for Medical Oncology (ESMO) guidelines suggested MMF 1 g twice daily for refractory immunotherapy-associated hepatitis [20]. However, there have been limited reports on the use of MMF in the context of immunotherapy-induced HLH, although there has been successful use in other secondary HLH [21]. Etoposide, despite being the proposed second-line treatment for refractory HLH after steroids [17], was therefore not chosen due to liver impairment [18]. Nevertheless, the effect of MMF as an immunosuppressant appeared effective alongside intravenous steroids and anakinra.

This combination of anakinra, MMF, and high-dose methylprednisolone achieved rapid clinical and biochemical resolution within 10 days, which aligned with adult HLH consensus guidelines advocating early, multi-agent immunosuppression to prevent relapse in hyperinflammatory syndromes [8]. This case highlights the importance of early detection and management of HLH in patients receiving immunotherapy, but also the additive benefits of steroids, anakinra and MMF.

## Conclusions

The combination of anakinra, mycophenolate mofetil (MMF), and high-dose methylprednisolone facilitated rapid clinical and biochemical remission within 10 days in this patient, aligning with adult hemophagocytic lymphohistiocytosis (HLH) consensus guidelines that recommend early, multi-agent immunosuppressive therapy to prevent relapse in hyperinflammatory conditions. This case underscores the critical importance of prompt recognition and treatment of HLH in patients undergoing immunotherapy. Furthermore, while our observations suggest a potential additive benefit of combining steroids, anakinra, and MMF in achieving disease resolution, future prospective studies and larger case series are required to formally establish the efficacy, optimal dosing and long-term safety of this multi-agent regimen.

## References

[1] Usmani GN, Woda BA, Newburger PE (2013). Advances in understanding the pathogenesis of HLH. Br J Haematol.

[2] Zhang L, Zhou J, Sokol L (2014). Hereditary and acquired hemophagocytic lymphohistiocytosis. Cancer Control.

[3] Larkin J, Chiarion-Sileni V, Gonzalez R (2019). Five-year survival with combined nivolumab and ipilimumab in advanced melanoma. N Engl J Med.

[4] Walmsley CS, Schoepflin Z, De Brabandt C, Rangachari D, Berwick S, Patell R (2025). Hemophagocytic lymphohistiocytosis associated with immune checkpoint inhibitor use: a review of the current knowledge and future directions. Blood Cells Mol Dis.

[5] Rivière S, Galicier L, Coppo P, Marzac C, Aumont C, Lambotte O, Fardet L (2014). Reactive hemophagocytic syndrome in adults: a retrospective analysis of 162 patients. Am J Med.

[6] Hagiwara S, Tanizaki J, Hayashi H (2024). Hemophagocytic lymphohistiocytosis induced by nivolumab/ipilimumab combination therapy: a case of lung adenocarcinoma that responded to early steroid pulse therapy. Cancer Rep (Hoboken).

[7] Gajagowni S, Wang E, Wang J, Campbell MT, Siddiqui BA (2025). Hemophagocytic lymphohistiocytosis (HLH) following immune checkpoint therapy (ICT). Case Rep Oncol Med.

[8] La Rosée P, Horne A, Hines M (2019). Recommendations for the management of hemophagocytic lymphohistiocytosis in adults. Blood.

[9] Filipovich AH (2009). Hemophagocytic lymphohistiocytosis (HLH) and related disorders. Hematology Am Soc Hematol Educ Program.

[10] (2026). Getting It Right First Time (GIRFT). Haemophagocytic Lymphohistiocytosis (HLH). Guidance on the diagnosis, treatment, management and governance. https://gettingitrightfirsttime.co.uk/wp-content/uploads/2026/04/HLH-Guide-UPDATED-April-2026.pdf.

[11] Eloseily EM, Weiser P, Crayne CB (2020). Benefit of anakinra in treating pediatric secondary hemophagocytic lymphohistiocytosis. Arthritis Rheumatol.

[12] (2026). Anakinra. LiverTox®: Clinical and Research Information on Drug-Induced Liver Injury.

[13] Azari AE, Stratton R, Singh A (2021). First case of hemophagocytic lymphohistiocytosis secondary to cabozantinib with checkpoint inhibitors. Rheumatology (Oxford).

[14] Abbasi AM, Shaikh MU, Shariq M, Arif MS, Arshad A, Raheem A, Ali N (2023). Outcome of patients with primary and secondary hemophagocytic lymphohistiocytosis: a retrospective analysis from a tertiary care center. Medicine (Baltimore).

[15] Xu Z, Li H, Yu X, Luo J, Zhang Z (2024). Clinical characterization of hemophagocytic lymphohistiocytosis caused by immune checkpoint inhibitors: a review of published cases. Hematology.

[16] Rajapakse P, Andanamala H (2022). Hemophagocytic lymphohistiocytosis secondary to immune checkpoint inhibitor therapy. World J Oncol.

[17] Carter SJ, Tattersall RS, Ramanan AV (2019). Macrophage activation syndrome in adults: recent advances in pathophysiology, diagnosis and treatment. Rheumatology (Oxford).

[18] Baverez C, Grall M, Gerfaud-Valentin M (2022). Anakinra for the treatment of hemophagocytic lymphohistiocytosis: 21 cases. J Clin Med.

[19] Charlesworth JE, Kavirayani A (2023). Intravenous anakinra for the treatment of haemophagocytic lymphohistiocytosis/macrophage activation syndrome: a systematic review. Eur J Haematol.

[20] Liu Z, Zhu Y, Xie H, Zou Z (2022). Immune-mediated hepatitis induced by immune checkpoint inhibitors: Current updates and future perspectives. Front Pharmacol.

[21] Saleh M, Hampel K, Gerth J, Merkelbach S, Monecke A, Mügge LO (2024). Combined immunosuppression with cyclosporin A, mycophenolate mofetil (MMF) and dexamethasone for activity control of recurrent secondary hemophagocytic lymphohistiocytosis (sHLH) with underlying systemic lupus erythematosus (SLE) [Article in German]. Inn Med (Heidelb).

